# Commentary: Consumer Reports of “Keto Flu” Associated With the Ketogenic Diet

**DOI:** 10.3389/fnut.2020.00113

**Published:** 2020-09-04

**Authors:** Miguel Sáenz de Pipaón, Katherine Flores-Rojas, Angel Gil, Mercedes Gil-Campos

**Affiliations:** ^1^Department of Neonatology, Hospital Universitario La Paz, Red de Salud Materno Infantil y Desarrollo - SAMID, Universidad Autónoma de Madrid, Madrid, Spain; ^2^Metabolism and Investigation Unit, Institute Maimónides of Biomedicine Investigation of Córdoba, Reina Sofia University Hospital, University of Córdoba, Córdoba, Spain; ^3^Department of Biochemistry and Molecular Biology II, Center of Biomedical Research, University of Granada, Institute of Nutrition and Food Technology “José Mataix,” Instituto de Investigación Biosanitaria IBS, Granada, Spain; ^4^CIBEROBN, Madrid, Spain

**Keywords:** ketogenic diet, blogs, adverse effects, scientific method, online forum

## Introduction

In recent years, interest in patient centered care has increased with the aim of improving patient experiences. An enormous amount of data describing health care experiences has been generated on social media including blogs, which are public forums were private experiences are described. Although there are advantages to using online forums to decrease research costs and catch a wider geographic or social area in research, it is important that they are not the single source of data used in an investigation.

Personal blogs and the opinions they contain offer insights into living experiences, including those connected with different illnesses. It has been suggested that patient experience of a disease can be inferred on a larger scale through automated textual analysis of health-related forums (1). However, using only this information, relevant clinical data could be missing with a selective publication and inadequate dissemination. If the data differs systematically from other published research that is based on scientific method, the results will be biased by an inaccurate assessment of the intervention effect. In publications based on non-professional blog information, no quality assessment tool exists. When patients access and share their experiences on online forums, they express a perception of their disease in a way that might be noticeably different from quality of life assessments with a more rigorous method of evaluation.

There is currently a lack of primary research on the limitations of social media in health communications among patients. There are some systematic reviews or meta-analysis that identify the uses, benefits, and limitations of social media in health communications (2, 3). The main recurring discussions in this literature outline the limitations of social media, examining quality concerns and the unreliable nature of this information. Importantly, automatic analysis cannot be conducted without a detailed knowledge of the subject area, advocating the need for more interdisciplinary research. Although some case samples of meta-analyses, not including gray literature or unpublished data, clearly overestimate treatment effects, quantifying this effect by considering all metaepidemiological studies implies minimal effects. In most health studies, the effects of excluding unpublished data are minimal, and the results are unaffected in the results. To have a real impact on science, more effective and reliable ways of locating and retrieving unpublished data and gray literature need to be developed, including the use of peer review and a lower risk of bias.

## Discussion

We read with interest the recent article on “Consumer Reports of “Keto Flu” Associated With the Ketogenic Diet” published in Frontiers in Nutrition (4), but would like to add some considerations. The article caused an immediate reaction in the media, with journalists discussing “flu” and its effects. However, these symptoms are based on information posted by people on social networks and this method and its conclusions are not reliable. This information was obtained from global internet forums that discussed keto flu, but which did not use or design a more objective validated method (i.e., questionnaire) to corroborate these opinions. It is interesting to consider, as the authors commented, that these discussion forums on health issues are a practical source for gathering information on patient experiences. However, the article's analysis and conclusion, that content from online forums provides new insights into the side effects of the Ketogenic Diet (KD), did not adhere to the criteria of a quantitative or qualitative scientific method. Gathering many people's experience of these symptoms does add information, but these sources do not provide evidence nor confirm the side effects of this treatment.

As the article commented, “the experiences of online forum users may not be representative of the larger group of people who follow the KD. However, the symptom patterns produced may indicate key lines of questioning for future survey-based approaches.” It is therefore necessary to work on these key lines of questioning before publishing, as the forums are unreliable. This information has not adjusted for factors such as the origin of the blog, the level of education, the type of KD diet, or if the information is manipulated, and therefore is not a complementary source in gathering clinical observations. It would be convenient to know more about the different people who participate in the forums, why they use a KD and if there were medical controls on their use diet.

The KD diet presents very precise indications for severe pathologies, with great side effects. Bostock et al. (4) indicate in their paper that the KD is often self-administered by patients, and that this could also be the situation for the users of online forums. There is limited evidence of its efficacy for conditions including weight loss, cognitive and memory enhancement, type II diabetes, cancer, neurological and psychiatric disorders. Keto flu may cluster a set of symptoms that appear when the body goes into ketosis with an electrolyte imbalance. The KD should be started with close monitoring by specialized medical professionals who can track hydration and the slow incorporation of adequate food to generate ketosis. When monitored, these effects can be avoided and are not interpreted as symptoms of the KD *per se*.

It is impossible to characterize the pattern, severity and time course of keto flu if it has not been previously described in scientific journals. Bostock et al. (4) did not include any specific references to literature about it. After reviewing the evidence from randomized controlled trials including 778 patients, this “flu” is not reported as being associated with KD (5). It is known that there are frequently side effects, as gastrointestinal, clearly related at starting the KD. At follow up appointments (between 2 and 16 months) some patients have reported infections (6–9). Even during childhood, the KD is an efficient and safe treatment and side effects such as nausea or constipation are early onset and not frequent (10). Moreover, “Flu KD” is neither a MeSh term nor a keyword related with this area of research. Symptoms of keto-induction were reported two decades ago (11), referred to in mainstream and gray literature as “keto-flu,” but these were not well-described in scientific literature (12). In recent years, other than the article by Bostock et al., there have been no publications.

## Author Contributions

All authors contributed to the search for scientific literature in this general commentary. This commentary advocates research that uses appropriate scientific methods, in this case related to the side effects of the ketogenic diet, and discusses opinions and experiences described in blogs, outlining that they do not contribute reliable scientific evidence.

## Conflict of Interest

The authors declare that the research was conducted in the absence of any commercial or financial relationships that could be construed as a potential conflict of interest.
